# Supplementary material for: EnzML: multi-label prediction of enzyme classes using InterPro signatures
Source: BMC Bioinformatics. 2012 Apr 25;13:61. doi: 10.1186/1471-2105-13-61 (PMC3483700; doi:10.1186/1471-2105-13-61)
Supplement: Addtional file 5 — The Java code to format the data files, evaluate and predict. The file enzml_java_code.tar.gz contains the Java code used to format database data to ARFF and XML formats, to execute cross and train-test (jackknife) evaluations and to record evaluation results to database. More information is included in the readme.txt file and the Javadoc files. The code can be used with a MySQL database. To use a different database software, other JDBC drivers might be required. [file 1471-2105-13-61-S5.gz › java_code/utils/doc/uk/ac/ed/inf/utils/webutils/ClientHttpRequestUtils.html]

ClientHttpRequestUtils


---


|  |  |  |
| --- | --- | --- |
| **Overview** \| **Package** \| **Class** \| **Use** \| **Tree** \| **Deprecated** \| **Index** \| **Help** | |  |
| PREV CLASS   **NEXT CLASS** | **FRAMES**    **NO FRAMES**     **All Classes** |  |
| SUMMARY: NESTED \| FIELD \| CONSTR \| METHOD | DETAIL: FIELD \| CONSTR \| METHOD |  |


---


## uk.ac.ed.inf.utils.webutils Class ClientHttpRequestUtils

```
java.lang.Object
  uk.ac.ed.inf.utils.webutils.ClientHttpRequestUtils
```

---

``` public class ClientHttpRequestUtils extends java.lang.Object ```

Title: Client HTTP Request class

Description: this class helps to send POST HTTP requests with various form
data, including files. Cookies can be added to be included in the request.

**Version:**
:   1.0

**Author:**
:   Vlad Patryshev

---

**Constructor Summary**

`ClientHttpRequestUtils(java.lang.String urlString)`
  
          Creates a new multipart POST HTTP request for a specified URL string

`ClientHttpRequestUtils(java.net.URL url)`
  
          Creates a new multipart POST HTTP request for a specified URL

`ClientHttpRequestUtils(java.net.URLConnection connection)`
  
          Creates a new multipart POST HTTP request on a freshly opened
URLConnection


| **Method Summary** | |
| --- | --- |
| `java.io.InputStream` | `post()`             posts the requests to the server, with all the cookies and parameters that were added |
| `java.io.InputStream` | `post(java.util.Map parameters)`             posts the requests to the server, with all the cookies and parameters that were added before (if any), and with parameters that are passed in the argument |
| `java.io.InputStream` | `post(java.util.Map cookies, java.util.Map parameters)`             posts the requests to the server, with all the cookies and parameters that were added before (if any), and with cookies and parameters that are passed in the arguments |
| `java.io.InputStream` | `post(java.lang.Object[] parameters)`             posts the requests to the server, with all the cookies and parameters that were added before (if any), and with parameters that are passed in the argument |
| `java.io.InputStream` | `post(java.lang.String[] cookies, java.lang.Object[] parameters)`             posts the requests to the server, with all the cookies and parameters that were added before (if any), and with cookies and parameters that are passed in the arguments |
| `java.io.InputStream` | `post(java.lang.String name, java.lang.Object value)`             post the POST request to the server, with the specified parameter |
| `java.io.InputStream` | `post(java.lang.String name1, java.lang.Object value1, java.lang.String name2, java.lang.Object value2)`             post the POST request to the server, with the specified parameters |
| `java.io.InputStream` | `post(java.lang.String name1, java.lang.Object value1, java.lang.String name2, java.lang.Object value2, java.lang.String name3, java.lang.Object value3)`             post the POST request to the server, with the specified parameters |
| `java.io.InputStream` | `post(java.lang.String name1, java.lang.Object value1, java.lang.String name2, java.lang.Object value2, java.lang.String name3, java.lang.Object value3, java.lang.String name4, java.lang.Object value4)`             post the POST request to the server, with the specified parameters |
| `static java.io.InputStream` | `post(java.net.URL url, java.util.Map parameters)`             posts a new request to specified URL, with parameters that are passed in the argument |
| `static java.io.InputStream` | `post(java.net.URL url, java.util.Map cookies, java.util.Map parameters)`             posts a new request to specified URL, with cookies and parameters that are passed in the argument |
| `static java.io.InputStream` | `post(java.net.URL url, java.lang.Object[] parameters)`             posts a new request to specified URL, with parameters that are passed in the argument |
| `static java.io.InputStream` | `post(java.net.URL url, java.lang.String[] cookies, java.lang.Object[] parameters)`             posts a new request to specified URL, with cookies and parameters that are passed in the argument |
| `static java.io.InputStream` | `post(java.net.URL url, java.lang.String name1, java.lang.Object value1)`             post the POST request specified URL, with the specified parameter |
| `static java.io.InputStream` | `post(java.net.URL url, java.lang.String name1, java.lang.Object value1, java.lang.String name2, java.lang.Object value2)`             post the POST request to specified URL, with the specified parameters |
| `static java.io.InputStream` | `post(java.net.URL url, java.lang.String name1, java.lang.Object value1, java.lang.String name2, java.lang.Object value2, java.lang.String name3, java.lang.Object value3)`             post the POST request to specified URL, with the specified parameters |
| `static java.io.InputStream` | `post(java.net.URL url, java.lang.String name1, java.lang.Object value1, java.lang.String name2, java.lang.Object value2, java.lang.String name3, java.lang.Object value3, java.lang.String name4, java.lang.Object value4)`             post the POST request to specified URL, with the specified parameters |
| `void` | `setCookie(java.lang.String name, java.lang.String value)`             adds a cookie to the requst |
| `void` | `setCookies(java.util.Map cookies)`             adds cookies to the request |
| `void` | `setCookies(java.lang.String[] cookies)`             adds cookies to the request |
| `void` | `setParameter(java.lang.String name, java.io.File file)`             adds a file parameter to the request |
| `void` | `setParameter(java.lang.String name, java.lang.Object object)`             adds a parameter to the request; if the parameter is a File, the file is uploaded, otherwise the string value of the parameter is passed in the request |
| `void` | `setParameter(java.lang.String name, java.lang.String value)`             adds a string parameter to the request |
| `void` | `setParameter(java.lang.String name, java.lang.String filename, java.io.InputStream is)`             adds a file parameter to the request |
| `void` | `setParameters(java.util.Map parameters)`             adds parameters to the request |
| `void` | `setParameters(java.lang.Object[] parameters)`             adds parameters to the request |

**Methods inherited from class java.lang.Object**

`equals, getClass, hashCode, notify, notifyAll, toString, wait, wait, wait`

**Constructor Detail**

### ClientHttpRequestUtils

```
public ClientHttpRequestUtils(java.lang.String urlString)
                       throws java.io.IOException
```

:   Creates a new multipart POST HTTP request for a specified URL string

    **Parameters:**: `urlString` - the string representation of the URL to send request to **Throws:**: `java.io.IOException`

---


### ClientHttpRequestUtils

```
public ClientHttpRequestUtils(java.net.URL url)
                       throws java.io.IOException
```

:   Creates a new multipart POST HTTP request for a specified URL

    **Parameters:**: `url` - the URL to send request to **Throws:**: `java.io.IOException`

---


### ClientHttpRequestUtils

```
public ClientHttpRequestUtils(java.net.URLConnection connection)
                       throws java.io.IOException
```

:   Creates a new multipart POST HTTP request on a freshly opened
    URLConnection

    **Parameters:**: `connection` - an already open URL connection **Throws:**: `java.io.IOException`


**Method Detail**

### post

```
public java.io.InputStream post()
                         throws java.io.IOException
```

:   posts the requests to the server, with all the cookies and parameters
    that were added

    :   **Returns:**: input stream with the server response **Throws:**: `java.io.IOException`

---


### post

```
public java.io.InputStream post(java.util.Map parameters)
                         throws java.io.IOException
```

:   posts the requests to the server, with all the cookies and parameters
    that were added before (if any), and with parameters that are passed in
    the argument

    :   **Parameters:**: `parameters` - request parameters **Returns:**: input stream with the server response **Throws:**: `java.io.IOException` **See Also:**: `setParameters`

---


### post

```
public java.io.InputStream post(java.util.Map cookies,
                                java.util.Map parameters)
                         throws java.io.IOException
```

:   posts the requests to the server, with all the cookies and parameters
    that were added before (if any), and with cookies and parameters that are
    passed in the arguments

    :   **Parameters:**: `cookies` - request cookies: `parameters` - request parameters **Returns:**: input stream with the server response **Throws:**: `java.io.IOException` **See Also:**: `setParameters`, `setCookies`

---


### post

```
public java.io.InputStream post(java.lang.Object[] parameters)
                         throws java.io.IOException
```

:   posts the requests to the server, with all the cookies and parameters
    that were added before (if any), and with parameters that are passed in
    the argument

    :   **Parameters:**: `parameters` - request parameters **Returns:**: input stream with the server response **Throws:**: `java.io.IOException` **See Also:**: `setParameters`

---


### post

```
public java.io.InputStream post(java.lang.String name,
                                java.lang.Object value)
                         throws java.io.IOException
```

:   post the POST request to the server, with the specified parameter

    :   **Parameters:**: `name` - parameter name: `value` - parameter value **Returns:**: input stream with the server response **Throws:**: `java.io.IOException` **See Also:**: `setParameter`

---


### post

```
public java.io.InputStream post(java.lang.String name1,
                                java.lang.Object value1,
                                java.lang.String name2,
                                java.lang.Object value2)
                         throws java.io.IOException
```

:   post the POST request to the server, with the specified parameters

    :   **Parameters:**: `name1` - first parameter name: `value1` - first parameter value: `name2` - second parameter name: `value2` - second parameter value **Returns:**: input stream with the server response **Throws:**: `java.io.IOException` **See Also:**: `setParameter`

---


### post

```
public java.io.InputStream post(java.lang.String name1,
                                java.lang.Object value1,
                                java.lang.String name2,
                                java.lang.Object value2,
                                java.lang.String name3,
                                java.lang.Object value3)
                         throws java.io.IOException
```

:   post the POST request to the server, with the specified parameters

    :   **Parameters:**: `name1` - first parameter name: `value1` - first parameter value: `name2` - second parameter name: `value2` - second parameter value: `name3` - third parameter name: `value3` - third parameter value **Returns:**: input stream with the server response **Throws:**: `java.io.IOException` **See Also:**: `setParameter`

---


### post

```
public java.io.InputStream post(java.lang.String name1,
                                java.lang.Object value1,
                                java.lang.String name2,
                                java.lang.Object value2,
                                java.lang.String name3,
                                java.lang.Object value3,
                                java.lang.String name4,
                                java.lang.Object value4)
                         throws java.io.IOException
```

:   post the POST request to the server, with the specified parameters

    :   **Parameters:**: `name1` - first parameter name: `value1` - first parameter value: `name2` - second parameter name: `value2` - second parameter value: `name3` - third parameter name: `value3` - third parameter value: `name4` - fourth parameter name: `value4` - fourth parameter value **Returns:**: input stream with the server response **Throws:**: `java.io.IOException` **See Also:**: `setParameter`

---


### post

```
public java.io.InputStream post(java.lang.String[] cookies,
                                java.lang.Object[] parameters)
                         throws java.io.IOException
```

:   posts the requests to the server, with all the cookies and parameters
    that were added before (if any), and with cookies and parameters that are
    passed in the arguments

    :   **Parameters:**: `cookies` - request cookies: `parameters` - request parameters **Returns:**: input stream with the server response **Throws:**: `java.io.IOException` **See Also:**: `setParameters`, `setCookies`

---


### setCookie

```
public void setCookie(java.lang.String name,
                      java.lang.String value)
               throws java.io.IOException
```

:   adds a cookie to the requst

    :   **Parameters:**: `name` - cookie name: `value` - cookie value **Throws:**: `java.io.IOException`

---


### setCookies

```
public void setCookies(java.util.Map cookies)
                throws java.io.IOException
```

:   adds cookies to the request

    :   **Parameters:**: `cookies` - the cookie "name-to-value" map **Throws:**: `java.io.IOException`

---


### setCookies

```
public void setCookies(java.lang.String[] cookies)
                throws java.io.IOException
```

:   adds cookies to the request

    :   **Parameters:**: `cookies` - array of cookie names and values (cookies[2\*i] is a name, cookies[2\*i + 1] is a value) **Throws:**: `java.io.IOException`

---


### setParameter

```
public void setParameter(java.lang.String name,
                         java.io.File file)
                  throws java.io.IOException
```

:   adds a file parameter to the request

    :   **Parameters:**: `name` - parameter name: `file` - the file to upload **Throws:**: `java.io.IOException`

---


### setParameter

```
public void setParameter(java.lang.String name,
                         java.lang.Object object)
                  throws java.io.IOException
```

:   adds a parameter to the request; if the parameter is a File, the file is
    uploaded, otherwise the string value of the parameter is passed in the
    request

    :   **Parameters:**: `name` - parameter name: `object` - parameter value, a File or anything else that can be stringified **Throws:**: `java.io.IOException`

---


### setParameter

```
public void setParameter(java.lang.String name,
                         java.lang.String value)
                  throws java.io.IOException
```

:   adds a string parameter to the request

    :   **Parameters:**: `name` - parameter name: `value` - parameter value **Throws:**: `java.io.IOException`

---


### setParameter

```
public void setParameter(java.lang.String name,
                         java.lang.String filename,
                         java.io.InputStream is)
                  throws java.io.IOException
```

:   adds a file parameter to the request

    :   **Parameters:**: `name` - parameter name: `filename` - the name of the file: `is` - input stream to read the contents of the file from **Throws:**: `java.io.IOException`

---


### setParameters

```
public void setParameters(java.util.Map parameters)
                   throws java.io.IOException
```

:   adds parameters to the request

    :   **Parameters:**: `parameters` - "name-to-value" map of parameters; if a value is a file, the file is uploaded, otherwise it is stringified and sent in the request **Throws:**: `java.io.IOException`

---


### setParameters

```
public void setParameters(java.lang.Object[] parameters)
                   throws java.io.IOException
```

:   adds parameters to the request

    :   **Parameters:**: `parameters` - array of parameter names and values (parameters[2\*i] is a name, parameters[2\*i + 1] is a value); if a value is a file, the file is uploaded, otherwise it is stringified and sent in the request **Throws:**: `java.io.IOException`

---


### post

```
public static java.io.InputStream post(java.net.URL url,
                                       java.util.Map parameters)
                                throws java.io.IOException
```

:   posts a new request to specified URL, with parameters that are passed in
    the argument

    :   **Parameters:**: `parameters` - request parameters **Returns:**: input stream with the server response **Throws:**: `java.io.IOException` **See Also:**: `setParameters`

---


### post

```
public static java.io.InputStream post(java.net.URL url,
                                       java.util.Map cookies,
                                       java.util.Map parameters)
                                throws java.io.IOException
```

:   posts a new request to specified URL, with cookies and parameters that
    are passed in the argument

    :   **Parameters:**: `cookies` - request cookies: `parameters` - request parameters: `url` - a URL **Returns:**: a stream **Throws:**: `java.io.IOException`

---


### post

```
public static java.io.InputStream post(java.net.URL url,
                                       java.lang.Object[] parameters)
                                throws java.io.IOException
```

:   posts a new request to specified URL, with parameters that are passed in
    the argument

    :   **Parameters:**: `parameters` - request parameters **Returns:**: input stream with the server response **Throws:**: `java.io.IOException` **See Also:**: `setParameters`

---


### post

```
public static java.io.InputStream post(java.net.URL url,
                                       java.lang.String name1,
                                       java.lang.Object value1)
                                throws java.io.IOException
```

:   post the POST request specified URL, with the specified parameter

    :   **Parameters:**: `name` - parameter name: `value` - parameter value **Returns:**: input stream with the server response **Throws:**: `java.io.IOException` **See Also:**: `setParameter`

---


### post

```
public static java.io.InputStream post(java.net.URL url,
                                       java.lang.String name1,
                                       java.lang.Object value1,
                                       java.lang.String name2,
                                       java.lang.Object value2)
                                throws java.io.IOException
```

:   post the POST request to specified URL, with the specified parameters

    :   **Parameters:**: `name1` - first parameter name: `value1` - first parameter value: `name2` - second parameter name: `value2` - second parameter value **Returns:**: input stream with the server response **Throws:**: `java.io.IOException` **See Also:**: `setParameter`

---


### post

```
public static java.io.InputStream post(java.net.URL url,
                                       java.lang.String name1,
                                       java.lang.Object value1,
                                       java.lang.String name2,
                                       java.lang.Object value2,
                                       java.lang.String name3,
                                       java.lang.Object value3)
                                throws java.io.IOException
```

:   post the POST request to specified URL, with the specified parameters

    :   **Parameters:**: `name1` - first parameter name: `value1` - first parameter value: `name2` - second parameter name: `value2` - second parameter value: `name3` - third parameter name: `value3` - third parameter value **Returns:**: input stream with the server response **Throws:**: `java.io.IOException` **See Also:**: `setParameter`

---


### post

```
public static java.io.InputStream post(java.net.URL url,
                                       java.lang.String name1,
                                       java.lang.Object value1,
                                       java.lang.String name2,
                                       java.lang.Object value2,
                                       java.lang.String name3,
                                       java.lang.Object value3,
                                       java.lang.String name4,
                                       java.lang.Object value4)
                                throws java.io.IOException
```

:   post the POST request to specified URL, with the specified parameters

    :   **Parameters:**: `name1` - first parameter name: `value1` - first parameter value: `name2` - second parameter name: `value2` - second parameter value: `name3` - third parameter name: `value3` - third parameter value: `name4` - fourth parameter name: `value4` - fourth parameter value **Returns:**: input stream with the server response **Throws:**: `java.io.IOException` **See Also:**: `setParameter`

---


### post

```
public static java.io.InputStream post(java.net.URL url,
                                       java.lang.String[] cookies,
                                       java.lang.Object[] parameters)
                                throws java.io.IOException
```

:   posts a new request to specified URL, with cookies and parameters that
    are passed in the argument

    :   **Parameters:**: `cookies` - request cookies: `parameters` - request parameters **Returns:**: input stream with the server response **Throws:**: `java.io.IOException` **See Also:**: `setCookies`, `setParameters`


---


|  |  |  |
| --- | --- | --- |
| **Overview** \| **Package** \| **Class** \| **Use** \| **Tree** \| **Deprecated** \| **Index** \| **Help** | |  |
| PREV CLASS   **NEXT CLASS** | **FRAMES**    **NO FRAMES**     **All Classes** |  |
| SUMMARY: NESTED \| FIELD \| CONSTR \| METHOD | DETAIL: FIELD \| CONSTR \| METHOD |  |


---
